# Supplementary material for: Anastomotic Leak in Ovarian Cancer Cytoreduction Surgery: A Systematic Review and Meta-Analysis
Source: Cancers (Basel). 2022 Nov 7;14(21):5464. doi: 10.3390/cancers14215464 (PMC9653973; doi:10.3390/cancers14215464)
Supplement: Supplementary file 1 [file cancers-14-05464-s001.zip › cancers-1971294-supplementary.pdf]

Supplementary material S1

Medline search strategy

1 Ovarian Neoplasms/
2 (ovarian tumo?r* or ovarian cancer* or ovarian neoplasm*).mp
3 Cytoreduction Surgical Procedures/
4 (Cytoreduction Surger* or Ovarian cytoreduction Surger*).mp
5 Debulking procedure*.mp
6 (Ovarian cancer surger* or primary advanced ovarian cancer surger*).mp
7 Anastomotic Leak/
8 Anastomotic leak*.mp
9 (Anastomosis Leak* or Anastomotic Leakage*).mp
10 Colon/
11 (Bowel* or Colon* or large bowel* or small bowel*).mp
12 (recto-sigmoid and colorectal resection).mp
13 (recto-sigmoid or colorectal resection).mp
14 Pelvic Exenteration/
15 pelvic exenteration*.mp
16 1 or 2
17 3 or 4 or 5 or 6
18 7 or 8 or 9
19 10 or 11 or 12 or 13 or 14 or 15
20 16 and 17 and 18 and 19

Embase search strategy

1 ovary cancer/
2 (ovarian tumo?r* or ovarian cancer* or ovarian neoplasm*)
3 cytoreductive surgery/
4 (Cytoreduction Surger* or Ovarian cytoreduction Surger*).mp
5 Debulking procedure*.mp
6 (Ovarian cancer surger* or primary advanced ovarian cancer surger*).mp
7 anastomosis leakage/
8 Anastomotic leak*.mp
9 Anastomosis Leak*.mp
10 colon/
11 intestine/
12 large intestine/
13 small intestine/
14 (Bowel* or Colon* or large bowel* or small bowel*).mp
15 (recto-sigmoid and colorectal resection).mp
16 (recto-sigmoid or colorectal resection).mp
17 pelvis exenteration/
18 Pelvic Exenteration*.mp
19 1 or 2
20 3 or 4 or 5 or 6

21 7 or 8 or 9
22 10 or 11 or 12 or 13 or 14 or 15 or 16 or 17 or 18
23 19 and 20 and 21 and 22
